# Impact of the implementation of new guidelines on the management of patients with HIV infection at an advanced HIV clinic in Kinshasa, Democratic Republic of Congo (DRC)

**DOI:** 10.1186/s12879-020-05470-0

**Published:** 2020-10-07

**Authors:** F. Mangana, L. D. Massaquoi, R. Moudachirou, R. Harrison, T. Kaluangila, G. Mucinya, N. Ntabugi, G. Van Cutsem, R. Burton, P. Isaakidis

**Affiliations:** 1 Médecins Sans Frontières, Kinshasa, Democratic Republic of Congo; 2 Médecins Sans Frontières, Conakry, Guinea; 3 Southern Africa Medical Unit, Médecins Sans Frontières, Cape Town, South Africa

**Keywords:** Advanced HIV, HIV clinical guideline, HIV infection, HIV clinical management, Sub-Saharan Africa

## Abstract

**Background:**

HIV continues to be the main determinant morbidity with high mortality rates in Sub-Saharan Africa, with a high number of patients being late presenters with advanced HIV. Clinical management of advanced HIV patients is thus complex and requires strict adherence to updated, empirical and simplified guidelines. The current study investigated the impact of the implementation of a new clinical guideline on the management of advanced HIV in Kinshasa, Democratic Republic of Congo (DRC).

**Methods:**

A retrospective analysis of routine clinical data of advanced HIV patients was conducted for the periods; February 2016 to March 2017, before implementation of new guidelines, and November 2017 to July 2018, after the implementation of new guidelines. Eligible patients were patients with CD4 < 200 cell/μl and presenting with at least 1 of 4 opportunistic infections. Patient files were reviewed by a medical doctor and a committee of 3 other doctors for congruence. Statistical significance was set at 0.05%.

**Results:**

Two hundred four and Two hundred thirty-one patients were eligible for inclusion before and after the implementation of new guidelines respectively. Sex and age distributions were similar for both periods, and median CD4 were 36 & 52 cell/μl, before and after the new guidelines implementation, respectively. 40.7% of patients had at least 1 missed/incorrect diagnosis before the new guidelines compared to 30% after new guidelines, *p* < 0.05. Clinical diagnosis for TB and toxoplasmosis were also much improved after the implementation of new guidelines. In addition, only 63% of patients had CD4 count test results before the new guidelines compared to 99% of patients after new guidelines. Death odds after the implementation of new guidelines were significantly lower than before new guidelines in a multivariate regression model that included patients CD4 count and 10 other covariates, *p* < 0.05.

**Conclusions:**

Simplification and implementation of a new and improved HIV clinical guideline coupled with the installation of laboratory equipment and point of care tests potentially helped reduce incorrect diagnosis and improve clinical outcomes of patients with advanced HIV. Regulating authorities should consider developing simplified versions of guidelines followed by the provision of basic diagnostic equipment to health centers.

## Background

Increased access to antiretroviral therapy (ART) has led to an overall decrease in HIV hospitalizations and subsequent mortality. However, mortality rates are still unacceptably high and most low-resourced countries are yet to meet targets set by the Joint United Nations Programme on HIV/AIDS (UNAIDS) back in 2014 [1]. .Compounding the problem is the fact that many patients still present late rendering the treatment of opportunistic infections (OIs) such as tuberculosis (TB), cryptococcal meningitis, Pneumocystis pneumonia (PCP) and non-TB pneumonia clinically complex [2]. The changing dynamics of the HIV epidemic including resistance to antivirals and antibiotics, change in demographic of infected patients, increase in the number of people living with HIV and the advancement of screening and testing techniques, has prompted calls for the review of existing guidelines so that they are comprehendible, accessible and adaptive to specific contexts and settings [3].

While generic guidelines have proven to be generally effective in the management of advanced HIV, reports and studies have shown that clinicians- especially those in resource limited settings- usually struggle to effectively incorporate guidelines into their everyday practice [4]. In countries with high HIV prevalence, algorithms and guidelines have been specifically adapted so that they are apt and responsive to their respective populations [5]. As such, HIV related hospitalizations and mortality in those countries have been on a steady decline [6–8]. On the other hand, in low HIV prevalence and low resource settings, most departments and agencies usually rely on the use of external clinical guides and algorithms for the management of HIV patients with OIs [9]. The use of these guidelines usually present a host of issues including mismatch between competencies of clinicians and proposed algorithms, lack of recommended resources, and complexity of language and/or presentation [3]. Therefore, over the years, health ministries, departments and organizations working in low prevalence and low resource settings have been reviewing existing clinical algorithms and guidelines so that they are specific and responsive to their context. As new guidelines are introduced, it is necessary for a review of those guidelines against previous guidelines to assess and measure their effectiveness and limitations in the real world clinical setting.

### Why this study?

Médecins Sans Frontières (MSF), with support from its Southern African Medical Unit (SAMU), has been working on reviewing and adapting existing advanced HIV clinical guidelines for use by it partners in low resource settings. These guidelines and algorithms are usually developed and adapted in consultation with other agencies and resources including the World Health Organization (WHO) and health ministries. The ultimate goal is to improve patient management in clinics and hospitals, thereby improving patient health outcomes. From April 2017 to November 2017, MSF-SAMU introduced and implemented a new guideline for use at “Centre Hospitalier De Kabinda” CHK in Kinshasa, Democratic Republic of Congo (DRC). After a successful implementation of the guideline, it was officially published in November, 2017. Both the previous and new guidelines focused on empirical treatment of OIs. However, the new guideline had a number of additions and revisions to help guide clinician decision making and improve patient outcomes. Changes made to the previous guideline included;

- Compilation of various clinical guideline documents into a single simplified guideline with explanatory texts and scenarios to help with diagnosis
- Improvement in the language and visual aesthetic of the guideline using images, colour codes, notepads, etc.
- Clarity in clinical diagnoses for opportunistic infections including clear guidelines and emphasis on empirical treatment
- Extensive training of clinicians on the new guidelines and support through HIV expert clinician visits from MSF-SAMU.
- Installation of advanced laboratory and point of care testing packages including GeneXpert®, TB-Mycobacterial Lipoarabinomannan (TB-Lam) and Serum Cryptococcal Antigen (CRAG) tests
- Periodically testing blood cultures of patients suspected of having OIs at external laboratories in Kinshasa.

The current study was conducted to evaluate the impact of the implementation of new guidelines on the management of patients hospitalized at the CHK in Kinshasa, DRC. In the past, results from routine annual reports showed a steady increase in mortality rates at the CHK from 2015 (24%), 2016 (28%), and the first quarter of 2017 (30%). A mortality audit conducted in July 2017 identified specific gaps in care. Diseases were underdiagnosed, and median times for the initiation of treatments were longer than recommended times. In the months following the implementation of algorithms from the new guidelines, significant decrease in mortality was observed at CHK. Although it would be difficult to determine the exact cause(s) for a change in mortality frequency using this study, nonetheless, this analysis will allow us to compare gaps in care before and after the implementation of new guidelines, and identify factors that may be associated with mortality at CHK during both periods.

### Specific objectives

Specifically, this study aimed to;

- Compare the accuracy of clinical diagnoses for OIs against existing algorithms
- Determine whether OIs diagnosed at admission are treated within recommended time frames
- Identify possible factors related to a change in mortality frequencies recorded during the study period.

## Method

### Type of study

The study is a retrospective, uncontrolled before and after cross-sectional study comparing clinical data of patients hospitalized at the CHK before and after the implementation of new clinical guidelines for the management of advanced HIV developed by MSF-SAMU [10].

### Study context

In Kinshasa, DRC, MSF has been operating one of the only dedicated advanced HIV referral clinic, the CHK. The clinic provides specialized care to advanced HIV patients and also provides support to partner and non-partner health centers in Kinshasa, DRC. The CHK was opened in 2002 providing only outpatient HIV services but expanded to providing inpatient care to advanced HIV patients in 2008. CHK today serves as a reference hospital and training center for MSF and its partners. The inpatient unit consists of a 41 bed hospital unit with an average of ~ 160 admissions per month and hospital stays averaging ~ 5 days. The outpatient unit follows an active cohort of ~ 1200 patients, and another 1500 patients from its partner health centers in Kinshasa. Although located in the Lingwala health zone of Kinshasa-DRC, the CHK admits HIV patients from all over Kinshasa and other provinces in DRC. The influx of patients is significant throughout the year with an inpatient bed occupancy rate of around 150% since 2017. Patients admitted at the CHK are mostly advanced HIV patients with a median CD4 of 76cells/μl [IQR, 21–200]. The hospital is currently the only advanced HIV clinic in Kinshasa, and for the context, provides a fairly “high technical platform”, with staff regularly receiving visits and technical support from HIV experts at the MSF-SAMU.

### Period of study, participants and sample size

Participants included hospitalized CHK patients who meet study eligibility criteria (see Eligibility criteria). For both periods (before and after the implementation of new guidelines), a total of at least 200 patients were included in the study. Sample sizes for both periods were estimated based on the annual number of hospitalized patients with complete clinic files, a 95% confidence interval and a 5% margin of error. Two time periods were identified for both periods; February 2016 to March 2017 for periods before implementation of new guidelines, and from November 2017 to July 2018 for periods after the implementation of new guidelines.

### Eligibility criteria

The following inclusion criteria were established a priori:

- Patients with a primary diagnoses of 1 of the following 4 OIs at admission: TB, PCP, Toxoplasmosis, non-TB pneumonia.
- a CD4 count at admission below 200 cell/μl
- and patients with complete clinic files

**Definition of correct, missed, incorrect and misclassified diagnosis:**

Correct Diagnosis: Diagnosis on file follows algorithm and meets the definition criteria as set out in the guidelines

Missed Diagnosis: Diagnosis not mentioned in patient’s file despite meeting diagnostic criteria established in the guideline

Incorrect Diagnosis: Does not meet diagnostic criteria set out in the guidelines

Misclassification of diagnosis (misclassification): Incorrectly labelling a diagnosis as either a primary or secondary diagnosis for patients with comorbidities/multimorbidities.

### Review of patient files

After selection of eligible patient folders by the principal investigator (FM), a committee of three experienced medical doctors at the CHK was set up to review patient files. The committee was headed by the medical team supervisor (TK). At least two out of the three committee members had to agree on a diagnosis before it could be determined as correct, incorrect, missed or misclassified. Patient files were reviewed against existing guidelines to determine accuracy of diagnosis, i.e. for each period, the guideline used at the time of diagnosis was used by the committee to determine accuracy of diagnosis. All committee members had used both guidelines, however, none of them had a direct role in the development of either guidelines.

### Data entry and analysis

Data was encoded into an excel database designed specifically for the study and checked for consistency and accuracy by cross referencing information in patients’ files to those available in the electronic management system at CHK. Files with inconsistent information that could not be verified were excluded. Univariate analysis was used to analyze and compare differences between continuous and categorical variables. Multivariate statistics was used to analyze mortality risk factors. Statistically significant threshold was set at 95% and data analysis was conducted using Microsoft Excel and STATA® software version 15.

## Results

### Characteristics of patients before and after the implementation of new protocol

**Fig. 1:**
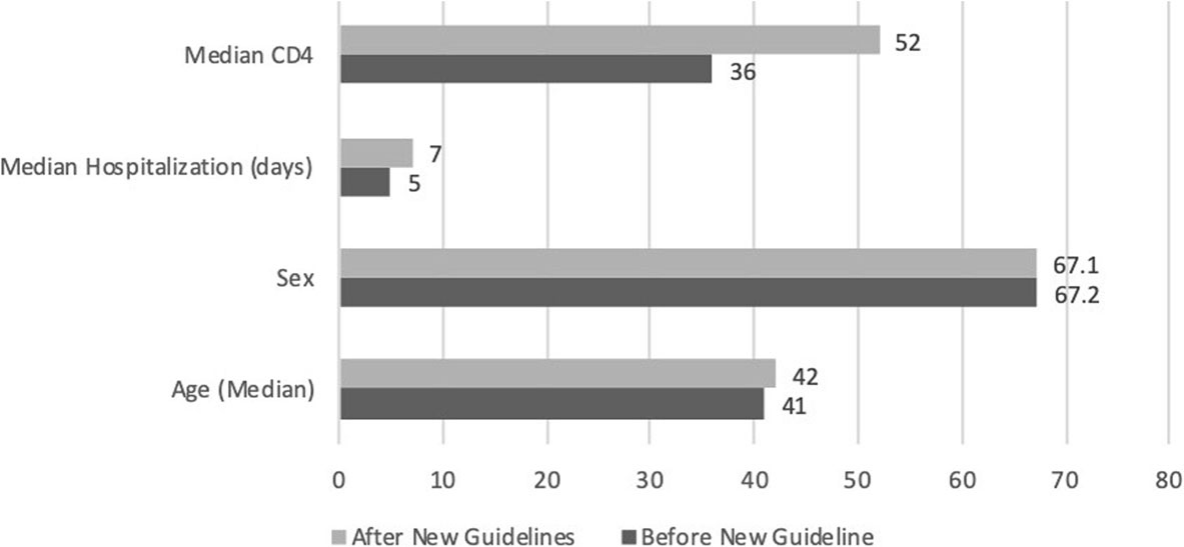
Patient characteristics before and after the implementation of new guideline

Patients included in analysis shared similar clinical backgrounds before and after the implementation of the new guidelines. Two hundred four and Two hundred thirty-one patients were included in the analysis before and after the implementation of new protocols respectively. Median age and sex distributions were similar for both periods, but median hospitalization days (after = 7[IQR2–10], before = 5[IQR4.4–13], *p* < 0.05%) and median CD4 counts (before = 36 cell/μl [IQR12–85], after = 52 cell/μl [IQR23–109], *p* < 0.05) were slightly higher after the implementation of new guidelines, see Fig. 1.

### Accuracy of clinical diagnosis

We reviewed patient files for 10 major OIs and clinical conditions; lung TB, central nervous and spinal (CNS) TB, non-CNS (TB), brain toxoplasmosis, PCP, Bacterial and Non-TB Pneumonia, Cryptococcal and bacterial meningitis, and bacterial septicemia. Among those reviewed, 40.7% (83/204) of patients had a missed/incorrect diagnosis before new empirical guidelines were implemented compared to 30% (60/231) of patients after the implementation of new guidelines, *p* < 0.05. Most (63%) patients with a missed/incorrect diagnosis after the implementation of new guidelines had only one missed diagnosis with only 36% having two or more missed diagnosis. A reversed pattern was observed in the period before the implementation of new guidelines, with most (64%) patients having at least 2 or more missed/incorrect diagnosis and only 36% having one missed/incorrect diagnosis, see Table 1.

**Table 1:** Patients with at least one missed/incorrect diagnosis

Number of missed diagnosis	Period	
	Before (%)	After (%)
One	30 (36)	38 (63)
Two	37 (45)	17 (28)
Three or more	16 (19)	05 (8)
Total	83	60*

* *p* < 0.05

### Accuracy of diagnosis for TB, toxoplasmosis, PCP, non-TB pneumonia

After the implementation of new guidelines, accuracy of diagnosis improved for TB, Toxoplasmosis, PCP and Non-TB pneumonia. However, statistically significant differences were only observed for TB and Toxoplasmosis diagnosis. Respectively, 92 and 82.7% of TB and Toxoplasmosis cases were correctly diagnosed after new guidelines were implemented compared to 63 and 62% of TB and Toxoplasmosis cases before the implementation of new guidelines, see Table 2. Among PCP diagnosis, 82% were correctly made after new guidelines compared to 65% before new guidelines (*p* > 0.05). For non-TB pneumonia, 54% of diagnosis were correctly made before new guidelines compared to 91.7% after implementation of new guidelines (p > 0.05). In addition, missed/incorrect or misclassification for all 4 diseases was lower after the implementation of new guidelines compared to before the implementation of new guidelines (*p* < 0.05).

**Table 2:** Common opportunistic infections and accuracy of diagnosis before and after the implementation of new guidelines

Type of diagnosis	Opportunistic infections (%)			
	Tuberculosis (TB)	Toxoplasmosis	Pneumocystis Pneumonia	Non-TB Pneumonia
Correct				
Before	47 (62.7)	43 (62.3)	36 (65.5)	7 (53.8)
After	106 (92.2)	62 (82.7)	61 (82.4)	11 (91.7)
Missed/Incorrect				
Before	19 (25.33)	12 (17.4)	6 (10.9)	2 (15.4)
After	2 (1.7)	9 (12)	6 (8.1)	1 (8.33)
Misclassification				
Before	9 (12)	14 (20.1)	13 (23.6)	4 (30.8)
After	7 (6.1)	4 (5)	7 (9.5)	0 (0)
Total				
Before	75	70	55	13
After	115	75	74	12
*p*-value*	< 0.01	0.01	0.06	0.08

*Fishers exact

### Multivariable logistic regression of mortality and danger signs

Mortality frequency for the periods after the implementation of new guidelines were lower than those before the implementation of new guidelines (before = 57.6 and 48.7% after), with statistically significant odds using a multivariate logistic regression model that included age, sex and nine danger signs, [OR = 0.60, CI (0.38–0.97), *p* < 0.05], see Table 3. Significant determinants of mortality included age, inability to walk at admission, and a Glasgow score of under 15, *p* < 0.05.

**Table 3:** Danger signs and mortality frequency

Related danger sign	Mortality (%)		Odds ratios	Confidence interval
	Before	After		
Age				
0–19	5.42	2.17	–	–
21–35	14.78	11.30	1.66	0.64–4.29
36–44	14.29	10.87	1.16	0.45–2.97
> 45	23.15	24.35	2.87*	1.13–7.29
CD4 < 50	9.85	6.96	0.97	0.60–1.56
Respiratory	24.14	17.83	0.77	0.46–1.28
Renal	33.66	29.13	1.08	0.67–1.74
Fever	51.72	43.91	1.45	0.68–3.11
Cardiovascular	53.47	48.70	0.81	0.49–1.35
Inability to walk	20.50	16.96	0.29*	0.18–0.48
Neurological	24.14	17.83	0.67	0.40–1.13
Dehydration	53.47	48.70	0.42	0.09–1.89
Glasgow< 15	61.22	26.15	0.84*	0.73–0.97
Sex				
Male	21.18	17.83	–	–
Female	36.45	30.87	0.69	0.41–1.15
Overall Mortality	57.64	48.70	0.60*	0.38–0.97

* *p* < 0.05

**Fig. 2:**
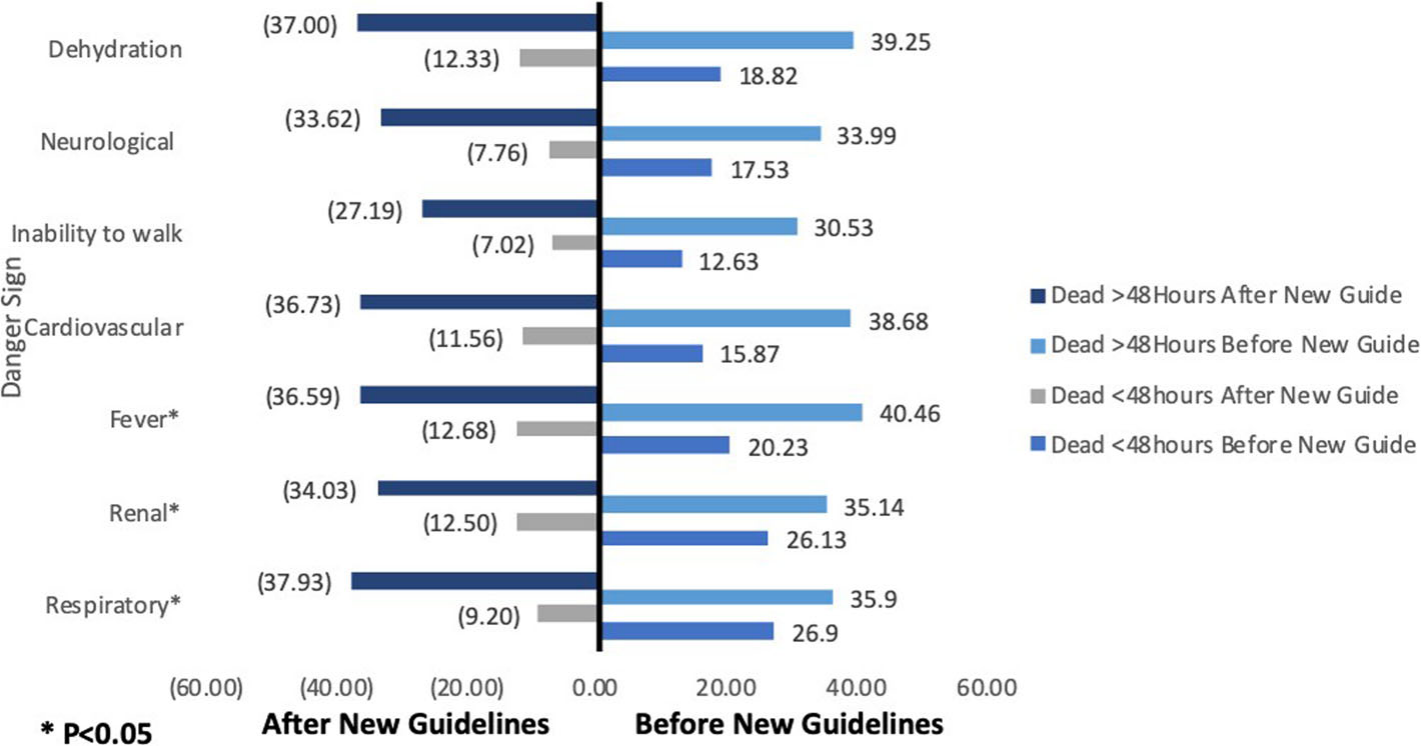
Danger signs and death frequency before and after 48 h

Mortality frequency before 48 h was 19.11% before the implementations of new guidelines compared to 13.7% after the implementation of new guidelines. Mortality after 48 h was also slightly lower in the periods after the implementation of new guidelines compared to before (36% compared to 38%). After the implementation of new guidelines, significant decreases in mortality before and after 48 h were observed for patients admitted with fever, renal failure and respiratory related danger signs. Mortality before and after 48 h among patients with danger signs such as extremely low CD4 count (</=50 cell/μl), dehydration, inability to work, cardiac and neurological related danger signs were also lower after the implementation of new guidelines compared to before (Fig. 2).

### Treatment turnaround times

During both periods, median treatment/turnaround times were similar for; when CD4 count results were received, Toxoplasmosis and PCP treatments were started after initial diagnosis, and when antibiotics were given to patients after prescription. However, treatment of TB after initial diagnosis improved from 2 days [IQR = 3] before new guidelines to 1 day [IQR = 1] after new guidelines, *p* < 0.05. In terms of when treatment was started after diagnosis, significant improvements were observed in the treatment of all three diseases in the first 24 h after the implementation of new guidelines compared to before. Also, after the implementation of new guidelines, nearly 80% of patients received their prescribed antibiotics within the first 24 h, an improvement of 24% compared to periods before the new guidelines were implemented (Table 4). In addition, after the implementation of new guidelines CD4 cell count results were only available for 63% of patients before the implementation of new guidelines compared to 99% after the implementation of new guidelines.

**Table 4:** Treatment/turnaround times

	Median (IQR)		0–24 h %		25–48 h %		> 48 h %	
	Before	After	Before	After	Before	After	Before	After
CD4 results	1 (2)	1 (1)	63.7	57.5	15.7	17.9	20.6	24.6
^a^TB*	2 (3)	1 (2.8)	36.8	61.1	20.2	9.9	42.9	29.01
^a^Toxoplasmosis*	1 (1)	1 (1)	56	69.6	12	10.9	32	9.6
^a^ PCP*	1 (2)	1 (2)	54.9	60.6	15.7	8.5	29.4	30.9
^b^Antibiotics Prescription	1 (1)	1 (1)	55.5	79.5	35.8	12.6	8.7	7.9

* *p* < 0.05

^a^Day treatment was started after diagnosis

^b^Day patient received the prescription from nurse

## Discussion

### Accuracy of clinical diagnosis

This study is among a few studies reporting on the effectiveness of clinical guidelines on the clinical management of OIs among advanced HIV patients. Similar studies have mostly focused on one or two principal morbidities, e.g. TB and/or pneumonia. Results from our study suggests that the introduction of an improved clinical guideline, coupled with clinician training and the installation of laboratory equipment and point of care tests potentially improved the accuracy of clinical diagnosis for the 10 OIs reviewed in this study. For the four principal OIs among advanced HIV patients, accuracy of diagnosis increased by at least 29.5, 20.4, 16.9 and 37.9% for TB, Toxoplasmosis, PCP and non-TB Pneumonia respectively, after the implementation of new guidelines. These results were not surprising as other reports and studies have also documented improvements in clinical outcomes when improved clinical guidelines are introduced to health care settings. For instance, a study by Opoka et al. (2019) on adherence to clinical guidelines and associated mortality noted lower mortality rates when clinical guidelines were effectively used by health care workers [11]. Another study by Dean et al. (2006) also reported that the implementation of a pneumonia treatment guideline was associated with improved patient’s clinical outcome [12]. Other studies on the effectiveness of new and improved guidelines for the management of sepsis, TB, and PCP also reported similar improvements in clinical decision making and patient outcomes [13]. The increase in accuracy of clinical diagnosis of OIs could also be attributed to the availability of advanced laboratory testing instruments as well as the introduction of point of care tests during the implementation of algorithms from clinical guidelines [14–16]. Shortly before the implementation of new clinical guidelines, the CHK introduced advanced laboratory and point of care testing packages including GeneXpert, TB-Lam and Serum Cryptococcal Antigen (CRAG) tests. The clinic also commenced periodically testing blood cultures of patients suspected of having OIs at external laboratories in Kinshasa. Thus, improvements observed in clinical diagnosis are thus consistent with expectations for accuracy of clinical diagnosis for OIs and improvement in patient outcomes [11, 12, 17].

### Mortality

Mortality frequency in this study far exceeds average frequency of deaths for patients admitted at CHK. Average death frequency (based on admissions) at CHK is usually between 26 and 28% compared to 57.64 and 48.70% before and after the implementation of new guidelines respectively. These results were however expected as the eligibility criteria for the study included patients who usually have high mortality rates at the clinic. For example, mortality frequency among advanced HIV patients presenting with TB as a primary diagnosis is ~ 60%, slightly above frequency of deaths recorded in the current study. We recognize that comparing mortality rates, especially for patients presenting with complex clinical pathologies can be problematic and may lead to false conclusions. Therefore, we are careful to directly relate reduced mortality rates to changes and recommendations made in the new guidelines alone, as there could be other contributing factors not accounted for in our analysis. That being said, other studies and reports have reported reduced mortality and increased survival when new and improved guidelines were introduced. Haynes et al. (2009), for example, in their study on the use of “surgical safety checklist to reduce morbidity and mortality in a global population”, reported that after the introduction of the checklist, there was an overall reduction in complications and subsequent deaths [18]. For our study specifically, possible contributions to the overall significant reduction in mortality could be attributed to the fact that the introduction of new clinical guidelines was also followed by the installation of advanced HIV laboratory testing equipment and of point of care test packages [19]. Furthermore, significant reductions in missed/incorrect diagnosis, especially for the four principal morbidities, could provide further explanation into why reduced mortality rates were observed. Globally, TB for example, accounts for 32% of deaths among advanced HIV patients, and this is mostly because 49% patients with HIV&TB comorbidities could not access appropriate healthcare [20]. It is therefore reasonable to suggest that an increase in accuracy of clinical diagnosis could potentially improve mortality rates among patients. Furthermore, the reduction of mortality among patients presenting with almost all danger signs after the implementation of new guidelines could also provide further explanation into the overall decrease in mortality after the implementation of new guidelines. Of note, significant odds were observed for patients who at admission could not walk, those with a Glasgow score of less than 15 and patients over 45 years of age. These results are consistent with results from other studies [21], and the WHO in their 2017 advanced HIV guideline for the management of advanced HIV, also included these as principal danger signs associated with high mortality among patients needing critical care [19].

### Treatment turnaround times

The main goal of the introduction of the new guidelines, point of care tests and the installation of laboratory equipment was to improve patient outcomes by providing clinicians with guidelines and resources that could improve their clinical decision making process in a timely manner. Results from this study showed that treatment turnaround times and antibiotics prescription were improved for the three main principal morbidities at CHK, especially for the first 24 h after admission. For most advanced HIV patients requiring emergency admissions, timely and appropriate care within the first 24 h after admission is critical. Guidelines for the empirical treatment of OIs recommend that patients generally receive antibiotics within 0–24 h after initial diagnosis [22, 23]. On the other hand, timing for when patients’ CD4 count test results were received by clinicians were better before the implementation of new guidelines. However, we must point out that a CD4 count point of care test, Abbott PIMA™, was introduced at CHK during the periods before the implementation of new guidelines. Another explanation for an increase in CD4 count results turnaround times after the implementation of new guidelines, could be associated to the problem of stock out of PIMA™ cartridges and FACSCount™ reagents, as there were more demands for these tests after the implementation of new guidelines. The issue of stock out in Kinshasa-DRC, was explored in a previous study on “Stockouts of HIV commodities in public health facilities in Kinshasa” [24]. Despite an increase in turnaround times for CD4 count test results after the implementation of new guidelines, almost all patients had a CD4 count done during admission (99%), compared to only 63% of patients before the implementation of new guidelines. Following current HIV protocol, this translates into improved triaging of patients and further appropriate testing including TB-LAM, CrAG, etc. [10, 19].

### Limitations

The current study was a retrospective cross-sectional study and as such is prone to limitations inherent to such studies [25, 26]. For example, most patient files before the implementation of new guideline had incomplete clinical information and so we had to use a more lengthy time period in order to reach achieve our target sample size. We tried to mitigate against some of these limitations by ensuring that we establish eligibility criteria a priori, cross check and validate data from patient files, ensure review by a panel of 3 medical doctors and a final independent review by the lead author (FM), and conduct multivariate statistics with appropriate covariates. In addition, attempting to compare clinical decisions using two time periods and different guidelines could be problematic in that it could be difficult to control or adjust for other confounding variables, e.g. level of training or experience of clinicians. Furthermore, even though clinical files were reviewed by a panel of three doctors, all of which had used both guidelines, there is always a possibility that some cases might have been inaccurately classified as correct, missed/incorrect or misclassified. Another potential bias could be the fact that the review committee was not blinded to both time periods, which could have been a potential source of bias. To mitigate against these, the study team took extra precautions to ensure that most, if not all, results are correct by ensuring that the committee reviewing clinical files were provided with specific algorithms contained in both guidelines for each OI under assessment, and ensuring that FM review all clinical files for a second time before encoding.

## Conclusion

The implementation of new guidelines improved the clinical management of patients with advanced HIV at the CHK, and could have potentially lead to a reduction in mortality at the hospital. Treatment turnaround times were also markedly improved especially for the commencement of treatments in the first 24 h. We therefore recommend that health ministries, departments and organizations consider developing or adapting HIV clinical guidelines so that they are apt and responsive to specific settings. Consideration should also be given to the provision of training for frontline health workers in the field on the use of and application of those guidelines. Lastly the provision of tests, e.g. point of care tests, and other clinical laboratory support could be helpful in the management of advanced HIV patients presenting with OIs.

## Data Availability

The datasets used and/or analyzed during the current study are available from the corresponding author on reasonable request.

## Abbreviations

AIDS: Acquired immunodeficiency syndrome
ART: Antiretroviral therapy
CHK: Centre Hospitalier De Kabinda
CNS: Central nervous and spinal
CRAG: Serum Cryptococcal Antigen
DRC: Democratic Republic of Congo
HIV: Human immunodeficiency virus
MSF: Médecins Sans Frontières
OIs: Opportunistic infections
PCP: Pneumocystis pneumonia
SAMU: Southern African Medical Unit
TB: Tuberculosis
TB-LAM: Tuberculosis mycobacterial lipoarabinomannan
UNAIDS: Joint United Nations Programme on HIV/AIDS
WHO: World Health Organization
